# Oscillometric blood pressure reference values of African full-term neonates in their first days postpartum

**Published:** 2009-12

**Authors:** Wilson E Sadoh, Samuel E Ibhanesebhor

**Affiliations:** University of Benin Teaching Hospital, University of Benin, Benin City, Nigeria; Wishaw General Hospital, College of Medicine and Veterinary Medicine, University of Edinburgh Medical School, Edinburgh, Scotland, UK

## Abstract

**Background:**

Knowing the normative blood pressure (BP) in a newborn baby is important in order to identify abnormal BP readings. This study was done to determine normative BP values of Nigerian newborns, using the 8100 Dinamap monitor.

**Methods:**

Consecutive full-term neonates delivered in a tertiary centre in Nigeria were recruited for the study. The babies’ systolic (SBP), diastolic (DBP) and mean arterial (MAP) blood pressures were measured within the first four days after birth.

**Results:**

A total of 473 babies were recruited for the study. The mean SBP, DBP and MAP readings on day 1 were 66.8 ± 7.7, 38.5 ± 6.3 and 47.9 ± 6.3 mmHg, respectively. The day 1 SBP of babies > 4 kg were significantly higher than those who weighed < 2.5 and 2.5–4 kg (*p* = 0.01, *p* = 0.05), respectively.

**Conclusion:**

This study provided current normative SBP, DBP and MAP values for Nigerian neonates. The BP readings compared with their Caucasian counterparts.

## Summary

Measurement of newborn blood pressure (BP) is important for cardiovascular evaluation. However, newborn BP is seldom measured, perhaps because of the cumbersome requirements for the diagnosis of hypertension in the newborn^1^,^2^ and the recommendation that universal screening of BP in neonates is unwarranted. 3 The non-availability of appropriate equipment for the measurement of neonatal BP may contribute to the attitude of healthcare providers not to measure BP.

Neonatal hypertension is increasingly being diagnosed in neonatal care units and occurs in 0.2 to 2.6% of neonatal intensive care survivals.^4^,^5^ The causes include umbilical artery catherisation,^6^ which is required for exchange blood transfusion, a procedure still commonly carried out for neonates with hyperbilirubinaemia in resource-poor settings.^7^ Other causes are cardiac anomalies, some medications and renal disorders.^6^,^8^ Early identification and management of hypertension is important.^6^,^8^ Similarly, diagnosed hypotension in the newborn may also require treatment.^9^ Normative BP values are required in order to diagnose hypertension or hypotension in neonates.

Most established normative BP values in neonates were either done on ill newborns in the neonatal units^10^ or on babies with a wide range of gestational ages.^10^ Also, most previous studies involving black subjects were on Africans living outside Africa. Since both genetic and environment factors may affect BP levels, it is important to determine the normative BP of African neonates in Africa.

To our knowledge, the last African BP study and perhaps the only one of its kind was done on Cameroonian neonates almost 20 years ago,^11^ and the BP was measured with a random-zero sphymomanometer (RZS). Although the RZS was designed to eliminate digit preference, this has not been shown to be the case.^12^ Furthermore, the RZS is being phased out of use as it contains mercury, a substance that can be injurious to health.^13^ The trend is towards the use of oscillometric devices which are easier to use, eliminate the difficulty with digit preference and are safe.

The present study therefore aimed to determine normative BP values of well, full-term Nigerian neonates in the postnatal wards of the University of Benin Teaching Hospital (UBTH), Benin City, Nigeria, using the 8100 Dinamap oscillometric device.

## Methods

Consecutive, full-term neonates born at or after 37 completed weeks of gestation, aged between one and four days, delivered at the UBTH, Benin City and admitted to the postnatal wards, were eligible for the study. Only those with normal APGAR scores were recruited. The babies with obvious congenital abnormalities, congenital heart diseases or neonates with any other morbidities requiring admission into neonatal units were excluded. Also excluded were babies of mothers with preeclampsia and diabetes.

Benin City is located in the tropical rainforest area in mid-western Nigeria. The annual delivery rate in the hospital is 2 000 babies. In the postnatal wards, the babies delivered vaginally were discharged within 48 hours, whereas babies delivered by caesarean section were discharged after five days, except when either mother or baby developed a complication.

The gestational age was determined by date (evaluated by the obstetricians) and early ultrasound where available. The babies were weighed using an infant weighing scale and their crown–heel length was taken in the supine position using a non-elastic tape. The BP was measured using a Dinamap 8100 monitor (Critikon, Tampa Fla) device. The BP measurements were taken by the author and three assistants who were all doctors and working in the neonatal unit. All investigators used the same protocol.

Each infant’s BP was measured one hour after feeds between 11:00 and 13:00, when the baby was asleep or awake and calm.^5^ Three sets each of systolic blood pressure (SBP), diastolic blood pressure (DBP) and mean arterial pressure (MAP) readings were obtained within three minutes of each other on the baby’s right arm. The average of the three readings was the determined BP value. The BP of any baby who cried during the procedure was discarded and the baby’s BP was determined the next day, except on the fifth day when the baby was excluded from the study. An appropriate critikon cuff (sizes 3 and 4), whose bladder width covered at least 40% of the circumference of the arm, and length covered 80% of the length of the arm, was used (length between the olecranon and acromion was determined).

The maternal age was obtained from the case notes.

Ethical approval for the work was obtained from the Ethics Committee of the UBTH, Benin City and verbal consent was obtained from the mothers of the babies, after detailed explanation of the study.

## Statistical analysis

Blood pressure readings, the baby’s weight, and the maternal age and weight were reported as mean ± SD (range). The BP readings on the first day postpartum in comparison with those of the second to fourth days (grouped) were presented in the fifth, 50th and 95th percentiles. The mean SBP, DBP and MAP values of the babies of different gestational maturity (AGA: appropriate for gestational age, SGA: small for gestational age and LGA: large for gestational age) and three birth-weight categories (< 2.5 kg, 2.5–4 kg and > 4 kg) were compared, using one-way ANOVA with Turkey-Kramer multiple comparison tests. The correlation of the BP readings with weight and gestational maturity was evaluated with the Pearson correlation test.

## Results

A total of 473 babies were recruited for the study, of which 244 (51.6%) were females and 229 (48.4%) were males. The majority (297 babies; 62.8%) were delivered vaginally, 125 (26.4%) were delivered by emergency caesarean section and 51 (10.8%) were delivered by elective caesarean section. The gestational age, mean maternal age and the mother and baby’s anthropometric measures are shown in Table 1. The distribution of babies according to gestational maturity and birth-weight categories are presented in Table 2. The gestational maturity was not determined in 25 (5.3%) babies. The majority (184; 38.9%) of the babies were studied on the first day after birth, 153 (32.4%) were examined on day 2, 80 (16.9%) on day 3, and 56 (11.8%) on day 4.

**Table 1:** Characteristics Of The Study Population

*Characteristics*	*Mean ± SD*	*Range*
Maternal age	29.9 ± 4.7	14 – 45
Maternal weight	71.0 ± 12.8	45 – 110
Gestational age	39.3 ± 1.4	37 – 43
Birth weight	3.210 ± 0.493	1.8 – 4.7
Baby’s length	49.3 ± 2.5	41 – 58

**Table 2:** Gestational Maturity And Birth Weight Distribution

*Characteristics*	*Number*	*%*
Gestational maturity		
AGA	378	79.9
LGA	26	5.5
SGA	44	9.3
Birth-weight categories		
< 2.5 kg	33	7.0
2.5–4.0 kg	402	85.0
> 4.0 kg	38	8.0

AGA: appropriate for gestational age, LGA: large for gestational age, SGA: small for gestational age. Gestational maturity was not determined for 25 babies.

There was no statistical difference in the mean SBP, MAP and DBP values (69.1 ± 8.4, 50.6 ± 7.8 and 40.6 ± 7.2 mmHg, respectively) of the male neonates compared to those of the females (69.4 ± 8.4, 50.8 ± 7.2 and 40.2 ± 6.2 mmHg, respectively). *P* values were 0.432, 0.823 and 0.562, respectively for SBP, MAP and DBP.

**Fig. 1.**
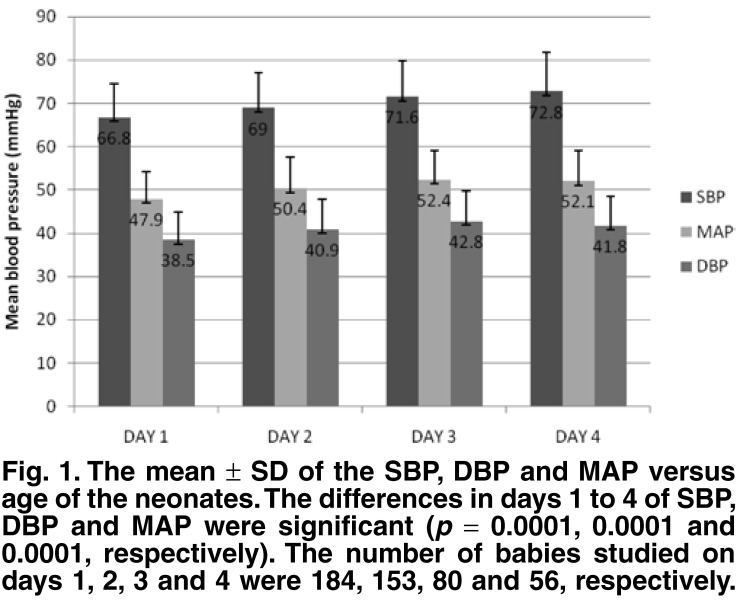
The mean ± SD of the SBP, DBP and MAP versus age of the neonates. The differences in days 1 to 4 of SBP, DBP and MAP were significant (*p* = 0.0001, 0.0001 and 0.0001, respectively). The number of babies studied on days 1, 2, 3 and 4 were 184, 153, 80 and 56, respectively

Mean SBP, DBP and MAP values on the different days of study are shown in Fig. 1. The SBP, DBP and MAP measurements progressively increased from day 1 to day 4. The mean SBP, DBP and MAP readings (66.8 ± 7.7, 38.5 ± 6.3, 47.9 ± 6.3 mmHg, respectively) on day 1 were significantly lower than those on day 2 (*p* < 0.01, *p* < 0.05 and *p* < 0.001, respectively). From days 2 to 4, the rise in SBP, DBP or MAP was not statistically significant (*p* > 0.05), nor was the difference in DBP and MAP between days 3 and 4 (*p* > 0.05). There was no statistically significant difference in the mean SBP, DBP and MAP of the females (69 ± 8, 40 ± 6, and 50 ± 7 mmHg, respectively) compared to those of the males (69 ± 8, 41 ± 7 and 50 ± 7 mmHg, respectively; *p* > 0.05 in all cases).

**Fig. 2.**
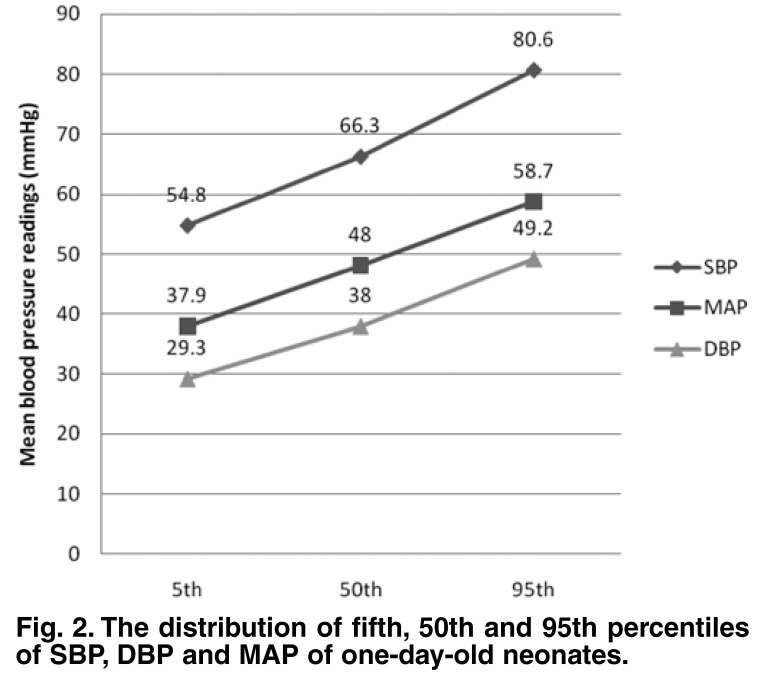
The distribution of fifth, 50th and 95th percentiles of SBP, DBP and MAP of one-day-old neonates.

**Fig. 3.**
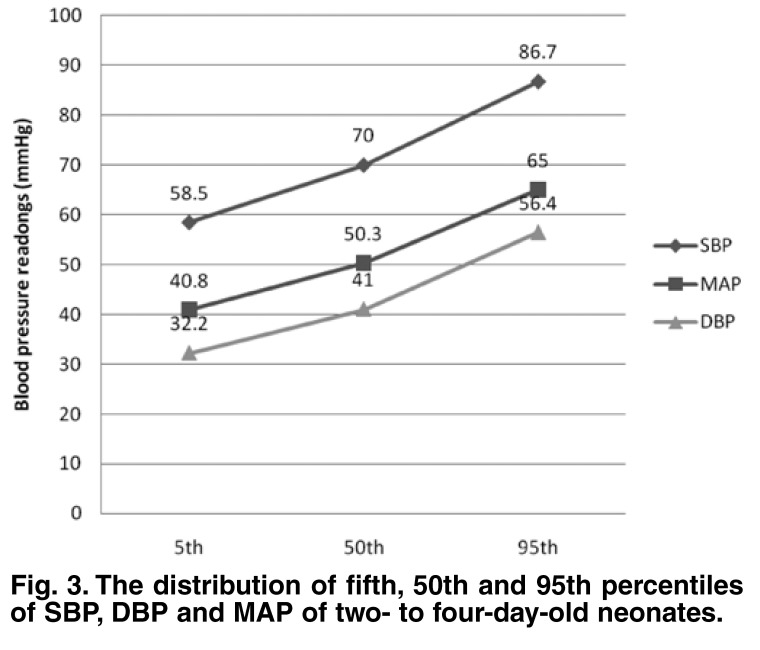
The distribution of fifth, 50th and 95th percentiles of SBP, DBP and MAP of two- to four-day-old neonates.

Since there was no statistically significant difference in SBP, DBP and MAP from day 2 to 4, these values were further analysed together as a group in comparison with day 1. The mean SBP of the group was 69.2 ± 8.3 (range: 48–108) mmHg, the mean DBP was 40.4 ± 6.8 (25–68) mmHg and the mean MAP was 50.0 ± 6.9 (33–78) mmHg. The fifth, 50th and 95th percentiles for SBP, DBP and MAP measured on day 1 and days 2 to 4 are shown in Figs 2 and 3.

**Fig. 4.**
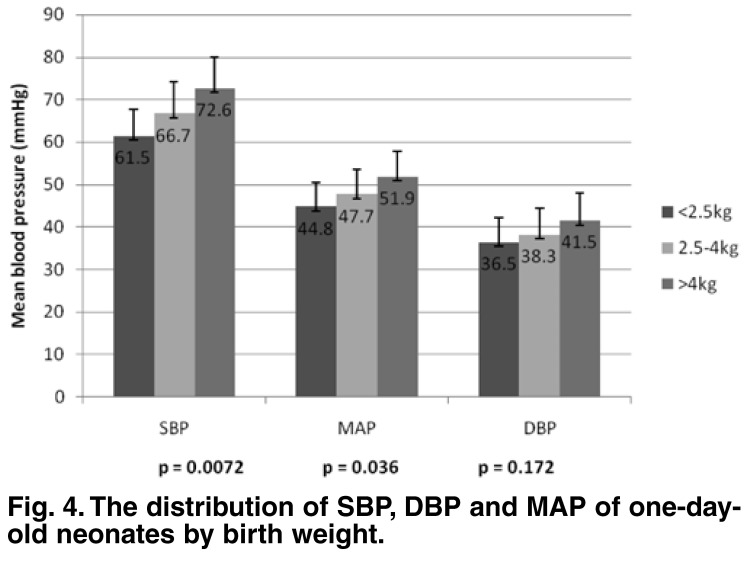
The distribution of SBP, DBP and MAP of one-day-old neonates by birth weight.

The mean SBP, DBP and MAP of day 1 and days 2 to 4 days were analysed with respect to birth-weight category and gestational maturity. The day 1 mean SBP of the babies who weighed > 4 kg was significantly higher than that for the babies < 2.5 kg (*p* ≤ 0.01) and for babies weighing 2.5–4 kg (*p* ≤ 0.05), whereas the mean MAP of babies who weighed > 4 kg was significantly higher than for babies who weighed < 2.5 kg (*p* < 0.05). The day 1 mean DBP was not significantly different with respect to the weight categories (*p* = 0.172) (Fig. 4).

**Fig. 5.**
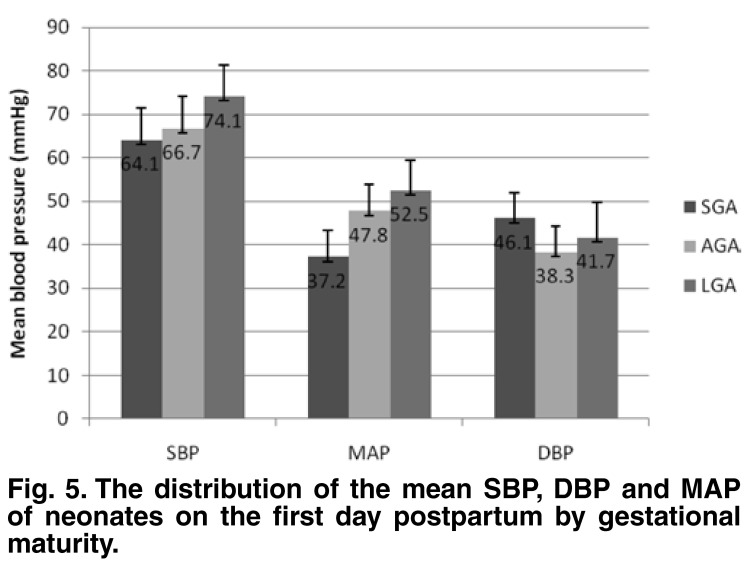
The distribution of the mean SBP, DBP and MAP of neonates on the first day postpartum by gestational maturity.

The mean SBP of LGA babies was significantly higher than that for AGA babies (*p* ≤ 0.05) and SGA babies (*p* = 0.01). Similarly, the mean MAP of LGA babies was significantly higher than that of SGA babies (*p* < 0.05). There was no difference in the mean DBP in the three categories (Fig. 5).

**Fig. 6.**
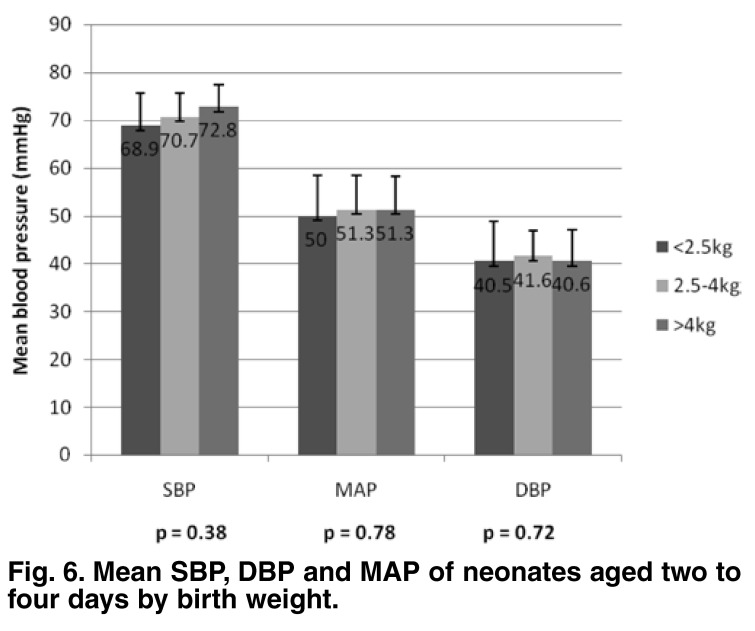
Mean SBP, DBP and MA P of neonates aged two to four days by birth weight.

**Fig. 7.**
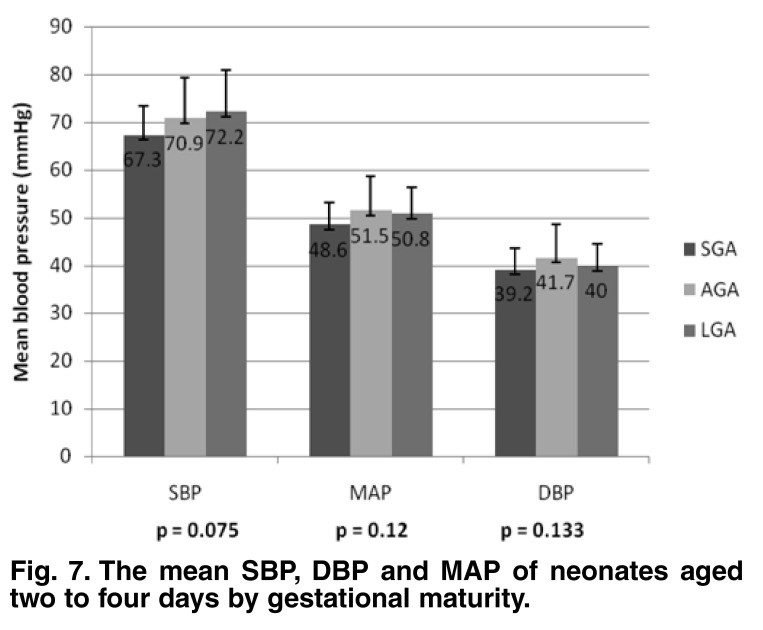
The mean SBP, DBP and MAP of neonates aged two to four days by gestational maturity.

The group (days 2–4) mean SBP, DBP and MAP values were not significantly different with regard to birth weight (*p* = 0.38, 0.72 and 0.78, respectively) (Fig. 6) or gestational maturity (*p* = 0.075, 0.133 and 0.12, respectively) (Fig. 7). There was a weak but significant correlation between birth weight and SBP, DBP and MAP values (*r* = 0.235, 0.137 and 0.185, respectively; *p* = 0.0001, 0.004 and 0.0001). The correlation between birth length and SBP and MAP were weak but significant (*r* = 0.110 and 0.098, respectively; *p* = 0.025 and 0.046, respectively).

## Discussion

In this study, current normative BP values of a healthy cohort of African neonates are provided. It is possibly the second such study after Youmbissi and co-workers^11^ studied 202 Cameroonian infants about two decades ago. In that study, only the SBP was measured with a random zero sphygmomanometer, longitudinally from birth to 12 months of age.

While comparison between the two studies may be difficult because of differences in equipment used, the SBP value at birth was 65.1 ± 1.30 mmHg (no range was given). This value is slightly lower than that of the present study (66.8 ± 7.7 mmHg). The small difference in SBP might be attributed to body weight, higher BP being associated with increased body weight, as has been shown by some workers.^14^ However, the mean birth weight of babies in the Cameroonian study (3.230 ± 0.500 kg) is comparable with that of babies in the present study (3.210 ± 0.493 kg). The different devices for measuring BP may have accounted for the difference, as higher BP values have previously been recorded by the Dinamap 8100 in comparison with a mercury sphygmomanometer.^15^,^16^

That only SBP was recorded in the Cameroonian study may have been due to limitations with the equipment used, and this is a drawback of the study. The fourth or fifth Korotkoff sounds are faint in the newborn and therefore difficult to auscultate using a mercury sphygmomanometer.^17^ The use of oscillometric devices eliminates this difficulty and provides systolic, diastolic and mean arterial blood pressure readings. The inability to follow up on the neonates in the present study beyond the first few days postpartum because of the hospital policy of early discharge was the main limitation of this study.

In a recent, similar study conducted on 406 neonates in Australia, using a different oscillometric device,^18^ although only median BP values were reported, the BP ranges were comparable on day 1 (46–94, 24–57 and 31–63 mmHg for SBP, DBP and MAP, respectively) and days 2 to 4. The characteristics of the neonates in the present study had some similarities with the Australian study in that the subjects were full-term normal babies evaluated in the postnatal ward, whose mothers had no confounding variables such as hypertension, diabetes, etc. In another study conducted by Park *et al.*^19^ in the United States, the normative oscillometric BP values for children five years and younger using a Dinamap 8100 were provided. The mean SBP of babies within the first week after birth was similar to that in the present study.

In contrast to the present study, most previous studies^2^,^10^ evaluated ill neonates in neonatal wards, some of whom were pre-term babies. However, the ranges of BP in these studies are comparable to the present study. This may suggest that normative BP values obtained from apparently healthy neonates may be applied to the ill newborn.

The significantly higher BP of LGA babies in comparison with SGA and AGA babies on the first day postpartum may be a reflection of the increase in BP with increasing birth weight, since the larger babies, over 4 kg, also had a significantly higher BP than those in the lower-weight categories. A weak but significant positive correlation between birth weight and SBP and MAP was also demonstrated in this study.

The relationship between birth weight and SBP on the first day postpartum in this study was in contrast to the Cameroonian^11^ and Brazilian^20^ studies where there was no correlation between birth weight and SBP on the first day. However, in the Cameroonian study, the SBP had a better correlation with the birth weight in older infants. This difference in SBP with respect to birth weight and gestational maturity was not replicated in BP values done at two to four days postpartum in this study. It is not clear why there was a significant difference in BP values in relation to birth weight in the one-day-old cohort, which was absent in the older neonates. Although it has been reported that babies born SGA tend to have a higher BP later in life,^21^ it appears that the major determining factor of blood pressure at birth, irrespective of the gestational maturity, is the body size.

## Conclusion

Normative SBP, DBP and MAP values are provided for African children using the Dinamap 8100 machine in this study. The BP ranges of this group of neonates compared with their Caucasian counterparts evaluated with comparable methods. There was a significant correlation between birth weight and SBP, DBP and MAP values on the first day postpartum. This difference seemed to disappear with age within the first week after birth.
